# Amendment to: populations in environments with a soft carrying capacity are eventually extinct

**DOI:** 10.1007/s00285-021-01624-z

**Published:** 2021-06-21

**Authors:** Peter Jagers, Sergei Zuyev

**Affiliations:** Department of Mathematical Sciences, Chalmers University of Technology and University of Gothenburg, SE-412 96 Gothenburg, Sweden

**Keywords:** Population dynamics, Extinction, Martingales, Stochastic stability, 92D25, 60G42, 60K40

## Abstract

This sharpens the result in the paper Jagers and Zuyev (J Math Biol 81:845–851, 2020): consider a population changing at discrete (but arbitrary and possibly random) time points, the conditional expected change, given the complete past population history being negative, whenever population size exceeds a carrying capacity. Further assume that there is an $\epsilon > 0$ such that the conditional probability of a population decrease at the next step, given the past, always exceeds $\epsilon$ if the population is not extinct but smaller than the carrying capacity. Then the population must die out.

## Three assumptions and one result

Denote population sizes, starting at time $\tau _0=0$, by $Z_0$, changing into $Z_1, Z_2, \ldots \in {\mathbb {N}}$ at subsequent time points $0< \tau _1 < \tau _2 \ldots$. Here ${\mathbb {N}}$ is the set of non-negative integers, and we make no assumptions about the times between changes. Let ${\mathscr {F}}_n$ be the sigma-algebra of all events up to and including the *n*-th change - i.e. really *all* events, not only population size changes - and introduce a *carrying capacity* $K>0$, the population size where reproduction turns conditionally subcritical. More precisely:

### Assumption 1

1 $$\begin{aligned} {{\,\mathrm{{{\mathbb {E}}}}\,}}[Z_{n+1}|{\mathscr {F}}_n] \le Z_n, \quad \mathrm {if}\ \ Z_n \ge K. \end{aligned}$$

Further,

### Assumption 2

There is no resurrection or immigration but, otherwise, a change is a change in population size:

2 $$\begin{aligned}&Z_n = 0 \Rightarrow Z_{n+1} =0, \end{aligned}$$

3 $$\begin{aligned}&Z_n > 0 \Rightarrow Z_{n+1} \ne Z_n. \end{aligned}$$

### Assumption 3

Non-extinct populations, smaller than the carrying capacity, run a definite risk of decreasing:

4 $$\begin{aligned} \exists \epsilon > 0; \forall n\in {\mathbb {N}}, 0< Z_n< K \Rightarrow {\mathbb {P}}(0\le Z_{n+1} < Z_n|{\mathscr {F}}_n] \ge \epsilon . \end{aligned}$$

Then:

### Theorem 1

Under the three assumptions given, the population must die out: with probability 1, $Z_n = 0$ eventually.

The original paper (Jagers and Zuyev 2020) had a stronger third assumption, *viz.* that, whatever the population history, there must be a definite, strictly positive risk that the population size decreases by exactly one unit at the next change. This is not unnatural and can be interpreted as a possibility that a change involves no reproduction but merely the death of one individual. But it turns out to be unnecessary.

## The proof

Like the original proof, this starts from stopping times $\nu _1, \nu _2, \ldots$ and $\mu _1, \mu _2, \ldots$, the former denoting the times of successive visits to the integer interval [0, *K*), the latter the subsequent first hittings of levels $\ge K$. More precisely,

$$\begin{aligned} \nu _1 := \inf \{n\in {\mathbb {N}}; Z_n < K\}, \end{aligned}$$

and for $$k=1,2, \ldots $$ ,

$$\begin{aligned} \mu _k := \inf \{n\in {\mathbb {N}}; n> \nu _k \text{ and } Z_n \ge K\}, \nu _{k+1} := \inf \{n\in {\mathbb {N}}; n > \mu _k \text{ and } Z_n < K\}. \end{aligned}$$

As was noted, $\nu _1<\infty$, whereas the $\mu _k$ constitute an increasing sequence, possibly hitting infinity. Clearly, $\nu _k< \infty , \mu _k=\infty$ means that the population dies out at or after $\nu _k$, without ever reaching *K* again. Also for any *k*, $\mu _k<\infty \Rightarrow \nu _{k+1} < \infty$. Proceeding like in the original paper, note that

$$\begin{aligned} Z_n \rightarrow 0 \Leftrightarrow \exists n \in {\mathbb {N}}; Z_n=0 \Leftrightarrow \exists k; \mu _k = \infty , \end{aligned}$$

and

$$\begin{aligned} {\mathbb {P}}( \exists k; \mu _k = \infty ) = \lim _{k \rightarrow \infty }{\mathbb {P}}(\mu _k = \infty ) = 1 - \lim _{k \rightarrow \infty }{\mathbb {P}}(\mu _k < \infty ). \end{aligned}$$

But

$$\begin{aligned} {\mathbb {P}}(\mu _k< \infty ) = {\mathbb {P}}(\mu _k< \infty , \nu _k< \infty ) = {{\,\mathrm{{{\mathbb {E}}}}\,}}[{\mathbb {P}}(\mu _k< \infty | {\mathscr {F}}_{\nu _k}) ; \nu _k < \infty .] \end{aligned}$$

For short, write

$$\begin{aligned} D_n := \{Z_n \le (Z_{n-1} -1)^+ \} \end{aligned}$$

for the event that the *n*-th change is a decrease, provided $Z_{n-1} >0$ (and of course the population remains extinct if $Z_{n-1} =0$). By Assumption 3, $Z_n < K$ implies that

$$\begin{aligned} {\mathbb {P}}(\cap _{j=1}^K D_{n+j}| {\mathscr {F}}_n)= & {} {{\,\mathrm{{{\mathbb {E}}}}\,}}[{\mathbb {P}}(D_{n+K} |{\mathscr {F}}_{n+K-1} ; \cap _{j=1}^{K-1} D_{n+j} | {\mathscr {F}}_n] \\\ge & {} \epsilon {\mathbb {P}}(\cap _{j=1}^{K-1} D_{n+j}| {\mathscr {F}}_n) \ge \ldots \ge \epsilon ^K .\end{aligned}$$

Since $Z_n < K$ implies that $Z_{n+K} =0$ on the set

$$\begin{aligned} \cap _{j=1}^K D_{n+j} , \end{aligned}$$

and the population size never crosses the carrying capacity, we can conclude that

$$\begin{aligned} {\mathbb {P}}(\mu _k = \infty )= & {} 1- {\mathbb {P}}(\mu _k< \infty ) \\\ge & {} 1- (1-\epsilon ^K) {\mathbb {P}}(\mu _{k-1} <\infty ) \ge \ldots \ge 1-(1-\epsilon ^K)^k \rightarrow 1. \end{aligned}$$

The theorem follows.
